# Frequent use of blood-saving measures in elective orthopaedic surgery: a 2012 Dutch blood management survey

**DOI:** 10.1186/1471-2474-14-230

**Published:** 2013-08-05

**Authors:** Veronique MA Voorn, Perla J Marang-van de Mheen, Manon M Wentink, Cynthia So-Osman, Thea PM Vliet Vlieland, Ankie WMM Koopman-van Gemert, Rob GHH Nelissen, Leti Van Bodegom-Vos

**Affiliations:** 1 Department of Medical Decision Making, Leiden University Medical Center, Albinusdreef 2, Leiden 2333, ZA the Netherlands; 2 Jon J van Rood Netherlands Center for Clinical Transfusion Research, Sanquin Research, Plesmanlaan 1-A, Leiden 2333, BZ the Netherlands; 3 Department of Orthopaedics, Leiden University Medical Center, Albinusdreef 2, Leiden 2333, ZA the Netherlands; 4 Department of Anesthesiology, Albert Schweitzer Hospital, Albert Schweitzerplaats 25, Dordrecht 3318, AT the Netherlands

**Keywords:** Blood management, Orthopedics, Arthroplasty, Survey

## Abstract

**Background:**

Blood loss in hip and knee arthroplasties may necessitate allogeneic blood transfusions. Different blood-saving measures (BSMs) were introduced to reduce these transfusions. Purpose of the present study was to assess the frequency of BSM use, stratified by type and hospital setting of orthopaedic departments in the Netherlands.

**Methods:**

An internet-based questionnaire was sent to all heads of orthopaedic departments of Dutch hospitals and private clinics (n = 99). Questions were asked on how often BSMs were used, reported on a 5-point Likert scale (never, almost never, regularly, almost always, always). In addition there were questions about discontinuation of anticoagulants preoperatively, the number of annually performed arthroplasties (size) and hospital setting.

**Results:**

The survey was completed by 81 (82%) departments. BSMs used frequently (regularly, almost always, always) were erythropoietine (EPO), with 55 (68%) departments being frequent users; acute normovolemic hemodilution, used frequently in 26 (32%) departments; cell saver in 25 (31%) and postoperative drainage and re-infusion in 56 (69%) departments. When compared by size, frequent EPO use was more common in large departments (with 22 (88%) large departments being frequent users versus 13 (63%) small departments and 16 (55%) intermediate departments, p = 0.03). No differences by size or type were observed for other BSMs.

**Conclusions:**

Compared with previous survey’s there is a tremendous increase in use of BSMs. EPO and autologous blood salvage techniques are the most often used modalities. Costs might be saved if use of non-cost-effective BSMs is stopped.

## Background

Blood loss in elective total hip and knee arthroplasties (THA and TKA) may necessitate allogeneic blood transfusions. Yet, allogeneic blood transfusions carry the risk of infections and noninfectious transfusion reactions [1,2]. Concerns about these risks have led to the development of blood-saving measures (BSMs) to reduce allogeneic blood transfusions. Many studies have been performed to assess the effectiveness and to a lesser extent the cost-effectiveness of various BSMs [3-9]. Results of reviews on this subject show that the effectiveness of the studied BSMs on allogeneic blood reduction and the accompanying costs vary largely among studies so that no firm conclusions on their use can be drawn [3-9]. Besides, the available reviews on BSMs show that the included studies had several limitations such as a retrospective design, small patient numbers and poor methodological quality [3-10]. In accordance with the latter in 2011 the Dutch transfusion guideline indicated that further research is needed to evaluate the cost-effectiveness of BSMs [11].

A multi-center randomized controlled trial with adequate power was therefore performed recently to investigate the cost-effectiveness of BSMs including preoperative erythropoietine (EPO), perioperative cell saver, and a postoperative drainage and re-infusion device (unwashed), in elective primary THA and TKA, using a restrictive transfusion trigger as described in the Dutch transfusion guideline [11]. It was shown that cell saver and postoperative drainage and re-infusion devices did neither result in a decreased mean red blood cell use, nor in a decrease in the proportion of transfused patients. Use of EPO showed a significant decrease in the proportion of transfused patients, but costs were considered too high. Adherence to a predefined uniform transfusion protocol was more than 95% in all participating hospitals. The conclusion of this study was thus that these three BSMs were not cost-effective in elective primary TKA and THA [12]. Annually, TKA are performed in about 20.000 patients and THA in about 26.000 patients [13], so that cost savings might be considerable if BSMs are de-implemented [14].

Previous surveys showed an increase in the use of BSMs between 1999 and 2007. A survey in 1999 showed that the proportion of Dutch hospitals using pharmacologic BSMs ranged from 0-2% and the use of non-pharmacologic BSMs ranged from 10-24% [15]. A more recent survey in 2007 showed that EPO and postoperative drainage and re-infusion were used in more than half of all Dutch orthopaedic departments [16]. However, these surveys did not distinguish between frequent and non-frequent use, nor in what hospital setting (university-, teaching-, general hospital or private clinic). This information is needed to focus on activities to abolish non cost-effective BSMs to those departments using BSMs frequently.

The aim of the present study was to assess the current frequency of BSM use, stratified by hospital setting and size of orthopaedic departments.

## Methods

An internet-based questionnaire was sent in January 2012 to all heads of orthopaedic departments of hospitals and private clinics within the Netherlands performing THA and TKA (n = 99). Hospitals with multiple locations were considered as a single hospital. Reminders were sent by email 2 weeks and 4 weeks after the first invitation, followed by a telephone call if necessary.

The study protocol has been presented to the Medical Ethical Committee of the Leiden University Medical Center. They declared ethical approval was not required under Dutch national law (CME 11/104).

### Questionnaire

The primary outcome measures of the questionnaire were the frequency of use of the following 11 BSMs: preoperative autologous donation, acute normovolemic hemodilution, intraoperative cell saver, postoperative drainage and re-infusion, EPO, tranexamic acid, desmopressin, epsilon aminocaproic acid, fibrin glue, platelet gel, and controlled hypotension. The frequency of use was reported on a 5-point Likert scale (never, almost never, regularly, almost always, always). The secondary outcome measures were if, and how many days prior to surgery, Coumadin derivatives, anti-platelet drugs and NSAIDs were stopped. This was used to assess whether there are associations between BSM use and stopping anticoagulant drugs preoperatively, as this might be part of blood management as well.

In addition, the questionnaire included questions about department characteristics such as hospital setting (university-, teaching-, general hospital or private clinic), size of department (number of primary and revision THA and TKA performed annually), and use of a restrictive transfusion protocol as described in the national transfusion guideline (yes/no) [11].

### Quality of care indicators

To test whether the transfusion rate in THA and TKA is associated with the frequency of BSM use, the results of this study were compared with self-reported allogeneic blood transfusion rates that are publicly available for all hospitals [17]. The number of patients with an ASA 1 or ASA2 classification [18] undergoing a total THA or TKA, and the number of these patients receiving an allogeneic blood transfusion are reported by all Dutch hospitals and private clinics.

### Analysis

First, all responders were analyzed together on frequency of BSM use to assess how often each BSM is used. Subsequently they were stratified into non-frequent users (never and almost never) and frequent users (regularly, almost always and always). It was tested whether frequent use varies among different hospital settings and size of departments (number of arthroplasties divided into tertiles). Differences between groups as well as associations between frequent use of BSMs and the preoperative continuation or discontinuation of Coumadin derivatives, anti-platelet drugs and NSAIDs were tested by chi-square tests. In case of expected cell counts less than five, the Fisher-exact test was used. Unpaired t-tests were used to test differences in transfusion rates.

For the analysis of the data the statistical software of SPSS v17.0 was used. P-values ≤ 0.05 were considered statistically significant in all analyses.

## Results

81 (82%) orthopaedic departments completed the questionnaire. The response rates did not differ between different hospital settings (*χ*^2^ = 0.74 with p = 0.86) (Table 1). The median number of annually performed arthroplasties among responding departments was 320 for THA (range 0–900) and 285 for TKA (range 35–900). In one clinic only TKAs were performed.

**Table 1:** Characteristics of orthopaedic departments responding to the questionnaire on blood saving measures

	**Total (n = 99)**	**Responders (n = 81)**	**% Responders of total**
University medical centre	8	5	63
Teaching hospital	28	24	86
General hospital	54	46	85
Private clinic	9	6	67

Table 2 shows the frequency of BSM use among the orthopaedic departments. Frequent use of preoperative EPO was seen in 55 (68%) departments and frequent use of autologous blood salvage was also common; with acute normovolemic hemodilution used frequent by 26 (32%) departments, cell saver by 25 (31%) and postoperative drainage and re-infusion by 56 (69%) departments. A few departments were frequent users of pre-operative autologous donation (3 (4%)), pharmacologic techniques other than EPO (range 0–10 (0-12%)) or the local techniques fibrin glue (3 (4%)) and platelet gel (1 (1%)).

**Table 2:** **Frequency of BSM use in Dutch orthopaedic departments of hospitals and private clinics** (**n** = **81**)

	***Non*-*frequent***			***Frequent***			
	**Never**	**Almost never**	**Total**	**Regularly**	**Almost always**	**Always**	**Total**
Preoperative Autologous Donation (%)	64 (79)	14 (17)	78 (96)	1 (1)	2 (3)	0	3 (4)
Acute Normovolemic Hemodilution (%)	35 (43)	20 (25)	55 (68)	14 (17)	8 (10)	4 (5)	26 (32)
Perioperative cell saver (%)	30 (37)	26 (32)	56 (69)	20 (25)	1 (1)	4 (5)	25 (31)
Postoperative Drainage and Re-infusion (%)	17 (21)	8 (10)	25 (31)	16 (20)	15 (19)	25 (31)	56 (69)
Erythropoietin (%)	8 (10)	18 (22)	26 (32)	37 (46)	9 (11)	9 (11)	55 (68)
Tranexamic acid (%)	58 (72)	13 (16)	71 (88)	4 (5)	3 (4)	3 (4)	10 (12)
Desmopressin (%)	70 (86)	11 (14)	81 (100)	0	0	0	0
Epsilon aminocaproic acid (%)	69 (85)	12 (15)	81 (100)	0	0	0	0
Fibrin glue (%)	67 (83)	11 (14)	78 (96)	3 (4)	0	0	3 (4)
Platelet gel (%)	74 (91)	6 (7)	80 (99)	0	0	1 (1)	1 (1)
Controlled hypotension (%)	32 (40)	22 (27)	54 (67)	17 (21)	6 (7)	4 (5)	27 (33)

When the departments were stratified by size, it was shown that frequent EPO use was more common in hospitals with large numbers of arthroplasties (Table 3). No significant differences were observed for the other BSMs. When stratified by hospital setting no significant differences were observed, although there is a trend (p = 0.07) that frequent EPO use was less common in university medical centers. Other observed trends were that frequent use of acute normovolemic hemodilution and controlled hypotension were more common in teaching hospitals and university medical centers (both p = 0.06) (Table 4).

**Table 3:** **Differences in frequent BSM use by number of arthroplasties per year** (**hospital size**)

	**Small <439 (n = 27)**	**Intermediate 439–720 (n = 29)**	**Large >720 (n = 25)**	**Total (n = 81)**	**P-value**
Preoperative Autologous Donation (%)	1 (4)	0	2 (8)	3 (4)	0.30
Acute Normovolemic Hemodilution (%)	8 (30)	8 (28)	10 (40)	26 (32)	0.59
Perioperative cell saver (%)	9 (33)	6 (21)	10 (40)	25 (31)	0.29
Postoperative Drainage and Re-infusion (%)	18 (67)	20 (69)	18 (72)	56 (69)	0.92
Erythropoietin (%)	17 (63)	16 (55)	22 (88)	55 (68)	0.03
Tranexamic acid (%)	4 (15)	1 (3)	5 (20)	10 (12)	0.16
Desmopressin (%)	0	0	0	0	
Epsilon aminocaproic acid (%)	0	0	0	0	
Fibrin glue (%)	0	2 (7)	1 (4)	3 (4)	0.39
Platelet gel (%)	0	1 (3)	0	1 (1)	0.40
Controlled hypotension (%)	12 (44)	5 (21)	9 (36)	27 (33)	0.16

**Table 4:** Differences in frequent BSM use by hospital setting

	**Private (n = 6)**	**General (n = 46)**	**Teaching (n = 24)**	**University (n = 5)**	**Total (n = 81)**	**P-value**
Preoperative Autologous Donation (%)	0 (0)	2 (4)	1 (4)	0 (0)	3 (4)	0.92
Acute Normovolemic Hemodilution (%)	0 (0)	12 (26)	11 (46)	3 (60)	26 (32)	0.06
Perioperative cell saver (%)	2 (33)	13 (28)	9 (38)	1 (20)	25 (31)	0.82
Postoperative Drainage and Re-infusion (%)	3 (50)	34 (74)	17 (71)	2 (40)	56 (69)	0.32
Erythropoietin (%)	3 (50)	33 (72)	18 (75)	1 (20)	55 (68)	0.07
Tranexamic acid (%)	1 (17)	4 (9)	4 (17)	1 (20)	10 (12)	0.72
Desmopressin (%)	0	0	0	0	0	
Epsilon aminocaproic acid (%)	0	0	0	0	0	
Fibrin glue (%)	0	2 (4)	1 (4)	0	3 (4)	0.92
Platelet gel (%)	0	0	1 (4)	0	1 (1)	0.49
Controlled hypotension (%)	2 (33)	10 (22)	12 (50)	3 (60)	27 (33)	0.06

Considering the effect of anticoagulant drugs on the frequency of BSM use, no effect was seen for the preoperative stopping of Coumadin derivatives and anti-platelet drugs (Data not shown). However, frequent EPO use was significantly more common in those hospitals that stopped NSAIDS before surgery (82% vs 60%, *χ*^2^ = 3.98 P =0.05).

Seventy-three (90%) of the respondents stated to use the national transfusion guideline [11]. Five departments use a different locally designed algorithm (extended version of the national transfusion guideline with advices for the use of BSMs) [19], the other 3 departments stated they do not have transfusions or they do not have a guideline. No associations were found between transfusion protocol and frequency of BSM use (data not shown).

When self-reported transfusion rates were compared with the frequency of BSM use, no differences in transfusion rate between frequent or non-frequent BSM use were found (Table 5).

**Table 5:** **Differences in percentage of transfused ASA 1 and ASA 2 patients in hospitals reporting frequent and non**-**frequent BSM use**

	**Non frequent use**	**Frequent use**	**CI**	**P-value**
Preoperative Autologous Donation (%)	5.6	4.3	−4.28 to 7.02	0.63
Acute Normovolemic Hemodilution (%)	6.3	4.2	−0.34 to 4.44	0.09
Perioperative cell saver (%)	5.4	6.0	−3.07 to 2.00	0.67
Postoperative Drainage and Re-infusion (%)	4.7	6.0	−3.75 to 1.16	0.30
Erythropoietin (%)	6.3	5.2	−1.34 to 3.46	0.38
Tranexamic acid (%)	5.4	7.6	−5.99 to 1.58	0.25
Desmopressin (%)	5.6			
Epsilon aminocaproic acid (%)	5.6			
Fibrin glue (%)	5.5	8.3	−8.41 to 2.83	0.33
Platelet gel (%)	5.7	0.0	−3.89 to 15.24	0.24
Controlled hypotension (%)	5.5	5.9	−2.81 to 2.02	0.74

## Discussion

The present study showed that BSMs are frequently used in TKA and THA, especially preoperative EPO use and postoperative drainage and re-infusion. Frequent EPO use is more common in hospitals with large numbers of arthroplasties, and a trend is found that frequent EPO use is less common in university medical centers. Frequent EPO use is significantly associated with stopping of NSAIDs before surgery. No differences were observed for the other BSMs.

A possible limitation of this study may be response bias if frequency of BSM use differs between responders and non-responders. Considering the high overall response rate (82%) and the fact that response did not differ per hospital setting, the effect of response bias is likely to be limited.

A second limitation of this study is the use of the subjective measure ‘number of BSMs used’. The use of this measure assumes that the respondents in our survey, i.e. the heads of orthopaedic departments of hospitals and private clinics within the Netherlands performing THA and TKA, have an adequate insight in the frequency of BSM use. Within the scope of this study it was not possible to verify the reliability of their reporting. However, given the evidence from the recent multi-centre randomized controlled trial of So-Osman et al. on the effectiveness of EPO, perioperative cell saver and postoperative drainage and re-infusion [12], the lack of an association between transfusion rates and BSM use seems plausible. Therefore the reported rates are likely to reflect actual practice.

Another limitation is the number of participating hospitals despite the high response rate in our survey (82%). Therefore, we were able to only detect large robust differences between frequent and non-frequent BSM users and we might have overlooked minor differences (i.e. type II error). However, it was not possible to further enlarge our study population, since we have approached all Dutch orthopaedic departments in The Netherlands for participation in our study.

A striking finding was that no association was found between frequent BSM use and the self-reported transfusion rates in ASA 1 and ASA 2 patients. This suggests that BSM use does not influence the transfusion rate so that these can be stopped without increasing this transfusion rate. As ASA 3 patients with more co-morbidity are more susceptible to receive allogeneic transfusions, it remains possible that these patients may benefit from BSMs. Given the restrictive transfusion policy used in the Netherlands, which has led to a significant drop in the number of blood transfusions [11,20], BSMs are probably used more frequently than strictly necessary on medical grounds.

The tremendous increase of BSM use over the past 13 years [7,8] in combination with the fact that EPO, cell saver, and postoperative drainage and re-infusion are not cost-effective [12] might give a huge cost reduction if these BSMs are abolished in clinical practice. The question remains why BSM use has increased so much over the past years, and whether barriers exist that prevent doctors to stop with these BSMs.

This survey is part of the ‘Leiden implementation study of blood management in hip and knee arthroplasties’ (LISBOA) which aims at designing an intervention to abolish non-cost-effective BSMs including EPO, perioperative cell saver and postoperative drainage and re-infusion from clinical practice. The present survey provides the target groups at which this intervention should be aimed. Frequent EPO use for instance is seen more commonly in departments with a large number of arthroplasties. The trend that frequent EPO use is less common in university medical centers is likely to be due to the fact that university medical centers don’t perform large numbers of arthroplasties annually. A possible explanation for this finding might be that the logistics behind EPO administration are complicated and time consuming, which can make it difficult for departments with smaller numbers of arthroplasties to use EPO. The association between frequent EPO use and stopping NSAIDs before surgery may be explained by extra caution taken in these hospitals to avoid blood transfusions. Interviews with involved medical specialists in a later phase of the LISBOA study will provide information about why departments choose for BSMs and which barriers they experience to stop using these BSMs. These results will also be taken into account in designing an intervention to abandon non cost-effective BSM use in clinical practice.

## Conclusions

In conclusion, this survey shows that BSMs are used frequently in TKA and THA in Dutch orthopaedic practice and its use has increased over the past years. The frequent use of EPO was observed particularly in large hospitals. Based on these findings, an intervention to abolish BSMs will be developed to arrive at a cost-effective blood transfusion policy. This will eventually lead to reduction of costs in perioperative orthopaedic care.

## Abbreviations

LISBOA: Leiden implementation study of bloodmanagment in hip and knee arthroplasties; BSMs: Blood saving measures; EPO: Erythropoietin; THA and TKA: Total hip arthroplasty and total knee arthroplasty; NSAIDs: Non-steroidal anti-inflammatory drugs; ASA: American society of anesthesiologists physical function score.

## Competing interests

All members of the LISBOA study group declare that there is no conflict of interest with any financial organization regarding the material discussed in the manuscript.

## Authors’ contributions

VV and LB designed the questionnaire. VV and MW conducted the questionnaire. VV, MW, LB and PM analyzed the data. VV drafted the manuscript. LB and PM coordinated the progress. LB, PM, CS, TVV, RN and AK assisted in critical revision of the questionnaire and manuscript. All authors read and approved the final manuscript.

## Pre-publication history

The pre-publication history for this paper can be accessed here:

http://www.biomedcentral.com/1471-2474/14/230/prepub
